# Pediatric infection and sepsis in five age subgroups: single-center registry

**DOI:** 10.1007/s10354-020-00787-6

**Published:** 2020-10-27

**Authors:** Michael M. Hermon, Theresa Etmayr, Jennifer Bettina Brandt, Kambis Sadeghi, Gudrun Burda, Johann Golej

**Affiliations:** 1 Department of Pediatrics, Division of Neonatology, Pediatric Intensive Care and Neuropediatrics, Medical University of Vienna, Währinger Gürtel 18–20, 1090 Vienna, Austria; 2 Department of Pediatrics, Ordensklinikum Linz, Barmherzige Schwestern Elisabethinen, Linz, Austria

**Keywords:** Pediatric sepsis, Severe infection, Pediatric intensive care, Extracorporeal membrane oxygenation, Multi organ failure, Pädiatrische Sepsis, Schwerwiegende Infektion, Pädiatrische Intensivmedizin, Extrakorporale Membranoxygenierung, Multiorganversagen

## Abstract

**Background:**

Sepsis is, worldwide, one of the leading causes of death among infants and children. Over the past two decades, mortality rates have declined due to advanced treatment options; however, the incidence of sepsis and septic shock is still on the rise in many hospital settings. The objective of this study was to evaluate the course of this disease in pediatric intensive care patients.

**Methods:**

An evaluation of pediatric patients in the intensive care unit diagnosed with infections or sepsis between 2005 and 2015 was performed via a retrospective exploratory data analysis.

**Results:**

During the observational period, 201 patients were diagnosed with infection or sepsis. The study population was divided into five age subgroups. The majority of patients were newborns, infants, and toddlers. Forty percent had sepsis; 6% had septic shock. Viral infection was the most prevalent (59%). The overall survival rate was 83%; newborns and adolescents had the lowest survival rates.

**Conclusion:**

With this registry, children divided into five age subgroups with infection or sepsis were evaluated and treatment strategies were examined. We have shown that our findings on children treated in our pediatric intensive care unit conform with current literature about pediatric sepsis. In addition to maintaining strict hygiene standards, optimal aspects of sepsis care should be stringently observed, such as the quick administration of empirical broad-spectrum antibiotics, initial adequate fluid resuscitation, and a reliable and frequent routine of source control.

## Abstract (de)

**Hintergrund:**

Sepsis ist weltweit eine der häufigsten Todesursachen bei Säuglingen und Kindern. In den letzten 2 Jahrzehnten sank die Sterblichkeitsrate durch verbesserte Behandlungsoptionen, die Inzidenz der Sepsis und des septischen Schocks nimmt jedoch weiterhin zu. Ziel dieser Studie war es, den Krankheitsverlauf bei pädiatrischen IntensivpatientInnen mit Sepsis oder Infektion zu beurteilen.

**Methodik:**

Es erfolgte eine retrospektive explorative Datenanalyse von pädiatrischen PatientInnen, die zwischen 2005 und 2015 aufgrund einer Infektion oder Sepsis auf der Intensivstation stationär aufgenommen worden waren.

**Ergebnisse:**

Während des Beobachtungszeitraums wurde bei 201 Patienten eine Infektion oder Sepsis diagnostiziert. Die Studienpopulation wurde in 5 Altersuntergruppen eingeteilt, die Mehrheit davon waren Neugeborene, Säuglinge und Kleinkinder. Eine Sepsis wurde bei 40 % und ein septischer Schock bei 6 % der PatientInnen diagnostiziert. Am häufigsten waren Virusinfektionen (59 %). Die Gesamtüberlebensrate betrug 83 %, dabei wiesen Neugeborene und Jugendliche die niedrigste Überlebensrate auf.

**Schlussfolgerung:**

Mit dieser Registeranalyse wurden die Daten von Kindern mit Infektion oder Sepsis evaluiert und Behandlungsstrategien untersucht. Es wurde dabei gezeigt, dass die vorliegenden Ergebnisse mit der aktuellen Literatur vergleichbar sind. Strenge Hygienestandards und optimale Sepsisversorgung sollten korrekt durchgeführt werden, wie z. B. rasche Verabreichung empirischer Breitbandantibiotika, entsprechendes Flüssigkeitsmanagement sowie zuverlässige und regelmäßige Laborkontrollen hinsichtlich der Infektionsquelle.

## Introduction

Worldwide, sepsis continues to be one of the most frequent conditions encountered in intensive care units (ICUs). While the incidence and prevalence of sepsis and septic shock is rising, the associated mortality rates are decreasing due to the ongoing development of diagnostic possibilities and treatment options [1, 2]. Sepsis continues to be one of the leading causes of ICU admission and places a substantial burden on healthcare costs [1–5]. It also remains a significant health problem for critically ill children and adult patients [6] and is one of the primary causes of death among infants and children [7, 8]. Furthermore, septic shock is one of the leading causes of multiple organ failure (MOF) and death in ICUs [9]. The high mortality of patients with multiple organ dysfunction remains a challenge for clinicians and deserves greater public health attention [4]. In addition, survivors often suffer from long-term consequences such as physical, psychological, or cognitive disabilities with social implications, requiring intensive and long-term healthcare [10]. The early recognition of and timely initial management of children with sepsis/septic shock is essential for achieving favorable outcomes. This, however, remains a major challenge for pediatric intensive care specialists as diagnostic and therapeutic recommendations are frequently derived from adult studies; comprehensive studies concerning the pathophysiology and management of pediatric sepsis are limited [6, 11, 12]. Therefore, the objective of this study was to evaluate the course of these pathological conditions in five subgroups of patients treated at our pediatric ICU (PICU).

## Methods

We performed a single-center, retrospective cohort study of critically ill children presenting to a multidisciplinary, tertiary pediatric intensive care unit (PICU). This eight-bed PICU serves a mixed population of medical, surgical, and trauma patients. We included all children 18 years or younger on admission who received care in the PICU between 2005 and 2015. Each hospitalization with a PICU admission was treated independently. All children with ICD-10 codes for infection, SIRS (systemic inflammatory response syndrome), sepsis, and septic shock were included in the study. Data were extracted from medical charts and laboratory files and analyzed from the day of PICU admission until discharge. The institutional ethics board of the Medical University of Vienna approved this study (ethic no. 1903/2015) and waived patient informed consent owing to the observational nature of the study. The study was performed in accordance with the Declaration of Helsinki.

The following data were extracted from patient files and analyzed: demographic data (age, gender, date of admission, weight, duration of PICU stay), outcome (survival rate), primary diagnosis (infection, sepsis, SIRS, septic shock, and multiorgan failure) according to the definition by Goldstein et al. [13], secondary diagnosis and comorbidities (genetic/congenital disorders, neurological disorders, immunodeficiency, metabolic disorders, and other disorders). Treatment options of study patients were noted: antibiotics, fluid resuscitation, vasopressors, steroids, immunoglobulins, insulin, renal replacement therapy, blood transfusion, extracorporeal membrane oxygenation (ECMO), therapeutic hypothermia, surfactant, and mechanical ventilation (days). The following physiological and laboratory data were collected on admission day, day 1, day 3, and day 7 of PICU stay: blood pressure, central venous pressure, pulse oximetry, body temperature, pH, base excess, lactate, C‑reactive protein (CRP), leucocytes, thrombocytes, and erythrocytes. Results of blood cultures and microbiology analyses were also collected. The patient data were collected in Excel (2016, Microsoft, Redmond Washington, USA) and the data analysis was performed using IBM SPSS Statistics version 24 for Windows (Armonk, NY, USA). Descriptive data analysis was used to describe the patients included in the registry. The chi-square and Kruskal–Wallis tests were used for statistical analysis. Results were accepted as statistically significant when *p* < 0.05. Data are presented as median with interquartile range (IQR), absolute number (*N*) of patients, or percentage (%).

## Results

Overall, 201 patients were included in this analysis. Demographic data of study patients are shown in Table 1.

**Table 1:** Demographic data and age subgroups

*Total number of patients, N (%)*	201 (100)
*Gender*	
Male	107 (53)
Female	94 (47)
*Age subgroups, N (%)*	
Newborn (0–30 days)	41 (20)
Infant (1–11 months)	62 (30)
Toddler (1–5 years)	45 (23)
Schoolchild (6–11 years)	37 (18)
Adolescent (12–18 years)	16 (9)

**Fig. 1:**
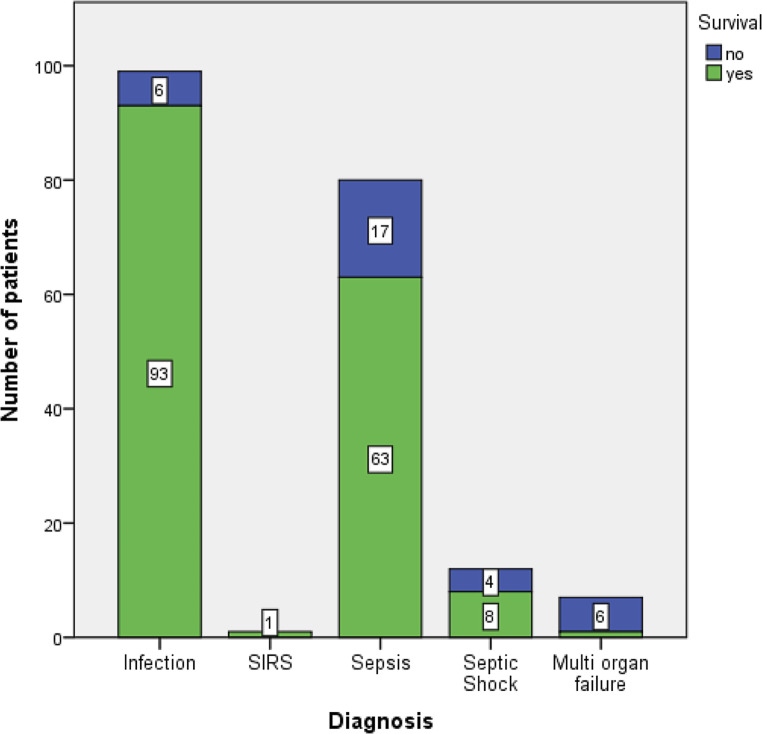
Survival rate in relation to primary diagnosis. *SIRS* systemic inflammatory response syndrome

The majority of study patients were newborns, infants, and toddlers. Schoolchildren and adolescents comprised 27% of all study patients. More than half of the study population were male (*n* = 107; 53%). A predominance of male patients was found in the age subgroups of newborns, infants, and schoolchildren. Total gender distribution between age subgroups was statistically significant (*p* = 0.028). The median age of male patients was 6.9 months and 15.9 months in female patients. There was a statistically significant difference in age between genders (*p* = 0.028). The primary diagnosis of study patients was divided as follows: infection 99 (50%), SIRS 1 (1%), sepsis 80 (40%), septic shock 12 (6%), and MOF 7 (3%) of all children. Distribution of primary diagnoses differed significantly between the five age subgroups (*p* < 0.001). Infection was the leading primary diagnosis among infants, toddlers, and schoolchildren. In the group of newborns and adolescents, however, the most frequent primary diagnosis was sepsis. Among the children with a primary diagnosis of infection, 59% were viral infections and 41% bacterial. Eighty-one percent of the viral infections were located in the lungs and 17% in the brain, while 2% were gastrointestinal (GI) infections. Thirty-nine percent of bacterial infections occurred in the lungs, 58% in the brain, and 3% were GI tract infection. More than half of the children 105 (54%) had underlying chronic diseases. The secondary diagnoses and comorbidities describing these diseases are listed in Table 2. There was no significant difference in gender distribution of secondary diagnoses (*p* = 0.759). 166 children (83%) survived their PICU stay. Table 2 shows the survival rates among the five different age subgroups. The lowest survival rates were seen in the newborn and adolescent subgroups. There were no significant differences in survival rates between the different age subgroups (*p* = 0.069). Fig. 1 shows survival rates in relation to the five primary diagnoses. The lowest survival rate of 16% occurred in children with MOF. There was a significant difference in survival rates between the different primary diagnoses (*p* < 0.001). Length of PICU stay showed a wide disparity; the median duration of PICU stay was 8 (4/15) days. The shortest PICU stay was 1 day; the longest 82 days. There was no significant difference in the duration of PICU stay between the different age subgroups or between the different diagnoses (*p* = 9.12; *p* = 6.955). The majority of children (83%) required mechanical ventilation during their PICU stay, with a median duration of 7.5 (4/14) days. The range of mechanical ventilation was 1 to 82 days. There was no significant difference in the duration of mechanical ventilation between the different age subgroups or between the different primary diagnoses.

**Table 2:** Survival rate within secondary diagnoses (comorbidities) and different age subgroups

	Survival		
	Yes *N* (%)	No *N* (%)	Total *N* (%)
*Secondary diagnoses (comorbidities)*			
None	77 (86)	13 (14)	90 (46)
Genetic/congenital disorder	48 (81)	11 (19)	59 (30)
Neurological disorder	14 (88)	2 (12)	16 (8)
Immune deficiency	3 (75)	1 (25)	4 (2)
Metabolic disorders	6 (55)	5 (45)	11 (6)
Other disorders	13 (87)	2 (13)	15 (8)
*Age groups*			
Newborn	10 (24)	31 (76)	41
Infant	10 (16)	52 (84)	62
Toddler	6 (13)	39 (87)	45
Schoolchild	3 (8)	34 (92)	37
Adolescent	6 (37)	10 (63)	16

Table 3 shows the different treatment options for all study patients (*n* = 201).

**Table 3:** Obtained treatment in the study population

Supportive care treatment	Yes/no (%)
Mechanical ventilation	83/17
Antibiotics	100/0
Fluid resuscitation	96/4
Corticosteroids	54/46
Vasopressors	51/49
Immunoglobulins	26/74
Insulin	15/85
Renal replacement therapy	20/80
ECMO	11/89
Surfactant	15/85
Blood transfusion	60/40
Therapeutic hypothermia	12/88

*ECMO* extracorporeal membrane oxygenation

All children received antibiotic therapy, the majority administered intravenously. The majority of children 84 (42%) were treated with antibiotics for 1 week. Fifteen patients (8%) received long-term antibiotic therapy for more than 21 days. The highest survival rate of 91% was observed in children undergoing antibiotic therapy for up to 2 weeks (8–14 days). However, only 9 children (60%) receiving >21 days of antibiotic therapy survived. In our study population a combination of broad-spectrum antibiotics was more frequently applied than a single-agent antibiotic treatment strategy. Antibiotics against anaerobic and atypical germs were never administered as single-agent antimicrobial therapies but rather in combination with other antibiotics. In 97 (48%) of the children, no blood cultures were taken. Blood cultures taken were negative in the majority of the patients. Positive blood cultures were found in 17% of all patients. Among the positive blood cultures, the most frequent pathogens were *Staphylococcus epidermidis* (*n* = 4), *Streptococcus pneumoniae* (*n* = 3), *Neisseria meningitides* group B (*n* = 2), beta-hemolytic *Streptococcus* (*n* = 2), and other bacteria (*n* = 7). In 86 patients no bacterial growth was detected. The majority of children (96%) received fluid resuscitation. Sixty-six percent received crystalloids, 5% colloids, and 29% a combination of both. In the septic children (*n* = 77) group, 61% received fluid resuscitation with crystalloids, 4% colloids, and 35% received both. Fifty-one percent of the children were treated with vasopressors. The majority of the patients receiving vasopressors and steroids were diagnosed with septic shock or MOF. Twenty-six percent of all study patients received immunoglobulins; the majority (22%) being IgM-enriched immunoglobulins with only a small number of patients (4%) receiving intravenous IgG. Immunoglobulins were most frequently used in sepsis, septic shock, or MOF patients. The use of immunoglobins was significantly different between the five primary diagnosis patient groups (*p* = 0.009). There was no survival benefit for patients receiving immunoglobulin therapy. ECMO was used in 21% [11] of all children. The median duration of ECMO was 8 (5/22) days. Only 9 children (43%) on ECMO survived. ECMO was most frequently used in patients diagnosed with sepsis or MOF (12 and 3, respectively). There were significant differences found in the use of ECMO between the different primary diagnoses (*p* = 0.014). Surfactant was applied in 15% of patients. Sixty percent of all patients obtained one or more blood transfusions. Therapeutic hypothermia was applied in 12 and 15% of all patients receiving insulin. In 20% of the children renal replacement therapy was used.

## Discussion

With this patient registry children with infections or sepsis were evaluated and treatment strategies examined. The aim was to get a precise description and evaluation of pediatric patients with infection or sepsis admitted to a PICU. During the 10-year study period, 201 patients were included in this patient registry. In our study population, we observed a higher number of male patients suffering from infection and/or sepsis (*n* = 107; 53%) than female patients (*n* = 94; 47%). This gender phenomenon revealing that males are more likely to be hospitalized with severe infections than females conforms with published studies [11, 12, 14].

Furthermore, in surgical patients, male gender can be an independent risk factor for developing severe infections [15]. As shown in Table 1, the study population was divided into five age subgroups: newborn, infant, toddler, schoolchild, and adolescent. Premature infants were excluded as they are primarily treated at neonatal intensive care units. Newborns and infants comprised more than 50% of all patients included in this registry. More than 70% were younger than 6 years old. Similar results were found in an epidemiology study of severe sepsis in children in the United States, where 48% of the study population were under 12 months of age [7]. The overall survival rate of patients included in the registry was 83%. The age group-related differences in PICU survival were not significantly different. As shown in Table 2, toddlers and schoolchildren had a high survival rate of over 85%. Lower survival rates were seen in newborns and infants. Adolescents had the lowest PICU survival of 63%. According to the literature, the highest mortality rates from sepsis are found in newborns and infants under 12 months of age. These age groups have the highest incidence of sepsis and the highest risk of sepsis-related deaths owing to low birth weight and prematurity [7, 11]. A possible explanation for the high mortality among adolescent septic patients is that the incidence of underlying chronic diseases—such as respiratory disorders, cardiac diseases, or malignancies with immune deficiencies—increase with age. More than half of our patients 105 (54%) had underlying chronic diseases. Survival in accordance with comorbidities is also shown in Table 2. The majority of adolescents hospitalized with sepsis had chronic disorders and thus had higher rates of sepsis-related mortality [9]. Another interesting aspect of our study was the analysis of survival rates in relation to the primary diagnoses (see Fig. 1). The study population was divided into the following primary diagnoses: infection, SIRS, sepsis, septic shock, and MOF. Fifty percent of the 201 patients in the registry were diagnosed with infections; another main diagnosis was sepsis (40%). Six percent of all patients were diagnosed with septic shock and 3% with MOF. As shown in Fig. 1, only one patient with SIRS was included in the registry. Because of the retrospective character of the study, it was not possible to ascertain whether this number is accurate; namely, that there was indeed only one SIRS patient during this 10-year study period. This underlines the problem of past sepsis definitions, as none were standardized. The various manifestations of sepsis and the multiple nonspecific definitions and terminologies made it difficult to define and categorize the septic patient. In 2005, the International Pediatric Consensus Conference (IPSCC) [13] proposed an age-adjusted definition for sepsis. Although the authors noted that their definition required improvement, pediatric specialists began using it in daily PICU practice worldwide [16, 17]. Regardless of the wide acceptance of the IPSCC definition in clinical settings, several studies have demonstrated its limitations [18, 19]. As shown in this large multicenter study [19], there was little consensus (46%) between the possible diagnosis of severe sepsis by the attending physician and the diagnosis according to the IPSCC definition. The SPROUT study investigators used a more liberal clinical approach. Since 2016, the Third International Consensus Definitions for Sepsis and Septic Shock (Sepsis-3) has been used, especially for adult patients, although SIRS is excluded in this definition [20]. Some authors question the applicability of the Sepsis‑3 definitions in children [21]. A study by Babay at al. published in 2005 analyzed bloodstream infections in pediatric patients and the results showed an overall mortality rate in their study population of 6% [22]. These results are similar to our findings. As shown by Goldstein at al., the highest risk of death occurred in children with MOF [13]. Mortality rates increase according to the severity and number of failed organs, reaching a value of around 50% when four or more organ systems are affected [7, 23]. High mortality rates in children with septic shock are most frequently associated with MOF [24–26]. All patients included in the registry received different forms of supportive care treatment (see Table 3). All the children obtained antibiotic therapy, the majority administered intravenously. The most frequently administered antimicrobials were broad-spectrum antibiotics. In most cases a combination of different antibiotics was necessary. This is in accordance with current international SSC (Surviving Sepsis Campaign) recommendations to administer an empiric first-line broad-spectrum antimicrobial therapy to cover most pathogens until the causative organisms are identified and a targeted therapy is feasible [27]. Another cornerstone of sepsis treatment is fluid resuscitation. The majority (96%) of the study population received fluid resuscitation (Table 3), with the most commonly administered being crystalloids. This conforms with the recommendation outlined in the SSC guidelines, which suggest that initial fluid resuscitation of sepsis-induced hypoperfusion crystalloid fluids should be given intravenously within the first 3 hours. There is no clear benefit of the administration of colloids compared to crystalloids in sepsis patients, thus crystalloid solutions should be the first line of choice for initial fluid resuscitation [27]. As reflected in Table 3, more than half of the patients in the registry were treated with corticosteroids and vasopressors. The majority of the patients receiving these substances were diagnosed with septic shock or MOF. The SSC guidelines note that vasopressor and corticosteroid support combined with volume resuscitation remains a standard line of therapy in septic shock. The physiological and positive effects of vasopressors in septic shock patients have been analyzed in numerous literature reviews [28–30].

Another treatment strategy we used for patients with infection or sepsis was immunoglobulin therapy. Twenty-six percent of the study population received immunoglobulin therapy. Administered immunoglobulins were primarily IgM-enriched IgG and, to a lesser extent, pure IgG immunoglobulin formulations. These were most frequently used in sepsis, septic shock, or MOF patients. Results of high-quality studies showed no statistically significant improvement of survival rates with use of immunoglobulins. At present, there is no evidence of survival benefit using immunoglobulins in sepsis patients and there is no recommendation in the SSC guidelines [27, 31–34]. Only a small number of children in the registry were treated with ECMO (*n* = 21); 11% of all study patients. Nine (43%) of the 21 ECMO patients survived. The median duration of ECMO was 8 (5/22) days. This therapy was most commonly used in children with sepsis (*n* = 12) or MOF (*n* = 3). ECMO was most frequently applied in newborns (*n* = 8), infants (*n* = 6), and adolescents (*n* = 3), which were also the age groups with the highest incidence of sepsis. Further evaluation of ECMO is needed to improve the efficacy of this treatment strategy [35]. In order to obtain bacteriological/virological surveillance of our patients, we use C‑reactive protein (CRP) as the first-line inflammatory biomarker. This biomarker is one of the most used in PICUs worldwide [18]. But there are known limitations in this diagnostic pathway, mainly due to the low sensitivity in differentiating cases of severe sepsis and common bacterial infections in an isolated measurement [36]. The most important role of CRP use is in the follow-up of sepsis children. A drop of more than 50% in CRP values on the fourth day of critical illness is associated with a good prognosis. No variations in CRP values indicate a poor therapeutic response to the antibiotic therapy [37]. Since 2017 we have been using procalcitonin and IL‑6, both having higher a diagnostic power for determining bacterial sepsis in children compared to CRP [38]. Due to the retrospective nature of our study there are a few limitations that should be pointed out: first, complete documentation of parameters is necessary for a coherent and sound interpretation. Relevant data, such as the daily infection surveillance, were unfortunately only partially or poorly documented. Second, it should be considered that the primary diagnoses may have been very discretional according to different healthcare providers, thus introducing a strong interpretation bias.

## Conclusion

In summary, our patient registry represents a highly valuable report of pediatric patients with sepsis or infection at a PICU and provides relevant feedback to intensive care clinicians in their daily work. In addition to strict hygiene standards, optimal aspects of sepsis care should be stringently performed, such as quick administration of empirical broad-spectrum antibiotics, initial adequate fluid resuscitation, and reliable and frequent routine of source control. By compiling and analyzing this patient registry, we have shown that our findings concerning children treated at the Medical University Hospital of Vienna conform to current literature about pediatric sepsis. Although data collection was carried out retrospectively, this patient registry could serve as a basis for its prospective continuation and extension. This would entail improved data collection and fewer of the aforementioned limitations, to more specifically analyze the impact of different treatment strategies in further research.
